# The inhibitory effects of plumbagin on the NF-қB pathway and CCL2 release in racially different triple-negative breast cancer cells

**DOI:** 10.1371/journal.pone.0201116

**Published:** 2018-07-30

**Authors:** Samia S. Messeha, Najla O. Zarmouh, Patricia Mendonca, Hayfaa Alwagdani, Malak G. Kolta, Karam F. A. Soliman

**Affiliations:** College of Pharmacy and Pharmaceutical Science, Florida A & M University, Tallahassee, Florida, United States of America; University of South Alabama Mitchell Cancer Institute, UNITED STATES

## Abstract

Breast cancer (BC) is the second leading cause of death among women in the US, and its subtype triple-negative BC (TNBC) is the most aggressive BC with poor prognosis. In the current study, we investigated the anticancer effects of the natural product plumbagin (PL) on racially different TNBC cells. The PL effects were examined in two TNBC cell lines: MDA-MB-231 (MM-231) and MDA-MB-468 (MM-468), representing Caucasian Americans and African Americans, respectively. The results obtained indicate that PL inhibited cell viability and cell proliferation and induced apoptosis in both cell lines. Notably, MM-468 cells were 5-fold more sensitive to PL than MM-231 cells were. Testing PL and Taxol^®^ showed the superiority of PL over Taxol^®^ as an antiproliferative agent in MM-468 cells. PL treatment resulted in an approximately 20-fold increase in caspase-3 activity with 3 μM PL in MM-468 cells compared with an approximately 3-fold activity increase in MM-231 cells with 8 μM PL. Moreover, the results indicate a higher sensitivity to PL in MM-468 cells than in MM-231 cells. The results also show that PL downregulated CCL2 cytokine expression in MM-468 cells by 30% compared to a 90% downregulation in MM-231 cells. The ELISA results confirmed the array data (35% vs. 75% downregulation in MM-468 and MM-231 cells, respectively). Moreover, PL significantly downregulated IL-6 and GM-CSF in the MM-231 cells. Indeed, PL repressed many NF-қB-regulated genes involved in the regulation of apoptosis, proliferation, invasion, and metastasis. The compound significantly downregulated the same genes (*BIRC3*, *CCL2*, *TLR2*, and *TNF*) in both types of cells. However, PL impacted five more genes in MM-231 cells, including *BCL2A1*, *ICAM1*, *IKBKE*, *IL1β*, and *LTA*. In conclusion, the data obtained in this study indicate that the quinone compound PL could be a novel cancer treatment for TNBC in African American women.

## Introduction

Breast cancer (BC) contributes to 23% of all cancer cases diagnosed in the US, making it the most common type and the second leading cause of cancer death among women [1]. In the United States, female BC cases are expected to rise and reach over 1.7 million with more than 600,000 death cases in 2018 [2]. The subtype TNBC is the most aggressive and metastatic BC that represents approximately 10–15% of all BC cases [3]. For BC treatments, hormone-based agents are targeting three characteristic receptors: estrogen (ER), progesterone (PR) or human epidermal growth factor (Her2/neu) [4, 5]. Although TNBC has initial higher response rates to a variety of chemotherapy agents, repeated exposure to chemotherapy can develop resistance to these agents [6], leading to poor prognosis and treatment failure. Moreover, TNBC aggressiveness is known to be more profound among African American patients (AA) than Caucasian American patients (CA) [7].

On the other hand, it is known that 15 to 20% of all cancer-related deaths worldwide are linked to inflammation [8]. The aggressiveness of TNBC is associated with the increased activity of nuclear factor-kappa B (NF-қB) transcription factor [9], which is thought to orchestrate the link between inflammation and cancer [10, 11]. NF-қB regulates genes involved in inflammation, cell- survival, apoptosis and proliferation in many solid tumors, including BC [12]. While NF-қB activation is immediate in normal cells, various oncogenic molecular modifications can lead to NF-қB pathway activation [13] that impacts the expression of different proinflammatory cytokines [14]. Moreover, cytokines such as IL-1β and TNF-α stimulated by NF-қB directly activate the NF-қB pathway to establish NF-қB autostimulation [15]. Indeed, inhibition of the NF-қB pathway can lead to significant downregulation of the inflammatory, procancerous events in several malignant tumors [16].

Tumors that arise at sites of chronic inflammation are characterized by the presence of macrophages, cytokines and chemokines [17]. In invasive BC, tumor-associated macrophages (TAMs) produce many proinflammatory cytokines, mainly, tumor-necrosis factor (TNF)-α [18, 19]. In BC patients, highly expressed TNF-α [18] stimulates proliferation of T47D cells of the human mammary gland [20] and upregulates several genes involved in cancer cell proliferation, invasion, and metastasis [21]. Additionally, an increase in TNF-α stimulates a release of the chemokine C-C Motif Ligand 2 (CCL2), which is also known as monocyte chemoattractant protein-1 (MCP-1) [22]. CCL2 is the most cancer-involved member of the CC chemokine family [23], and its high expression in the tumor tissue promotes tumorigenesis and metastasis [24]. Moreover, CCL2 suppression is associated with reduced tumor aggressiveness in BC [25].

Many studies show the anticancer and anti-inflammatory properties of plumbagin (PL), the main active constituent of *Plumbago zeylanica* roots. The plant roots have been used in India for many centuries in treating skin diseases, diarrhea, dyspepsia, piles, anasarca, plague, leprosy, urinary tract infections, scabies, and ulcers [26]. Moreover, the plant was found to have neuroprotective, hepatoprotective, antiatherogenic, and cardiotonic properties [27]. PL is found in other medicinal plants belonging to the Plumbaginaceae, Droseraceae, and Ebenaceae families [28]. Recent reports indicate the use of PL in treating diseases that are associated with inflammation, such as rheumatoid arthritis [29]. Our previous study indicates that PL has a potent anti-inflammatory effect in BV-2 microglia cells [30]. The PL anticancer properties have been studied in many cancers including breast [31], prostate [32, 33], and ovarian [34] cancers. The anticancer property of PL was also reported in pancreatic [35], lung [36], cervical [37, 38], and brain [28] cancers.

Therefore, we selected two human TNBC cell lines, MDA-MB-231 (MM-231) and MDA-MB-468 (MM-468), as associated with CA and AA races, respectively [39]. We hypothesized that the NF-қB pathway is involved in the PL-repressing effect of CCL2 and may also impact NF-қB-regulated genes which orchestrate the intra- and inter-cellular anticancer action.

## Results

**Fig 1:**
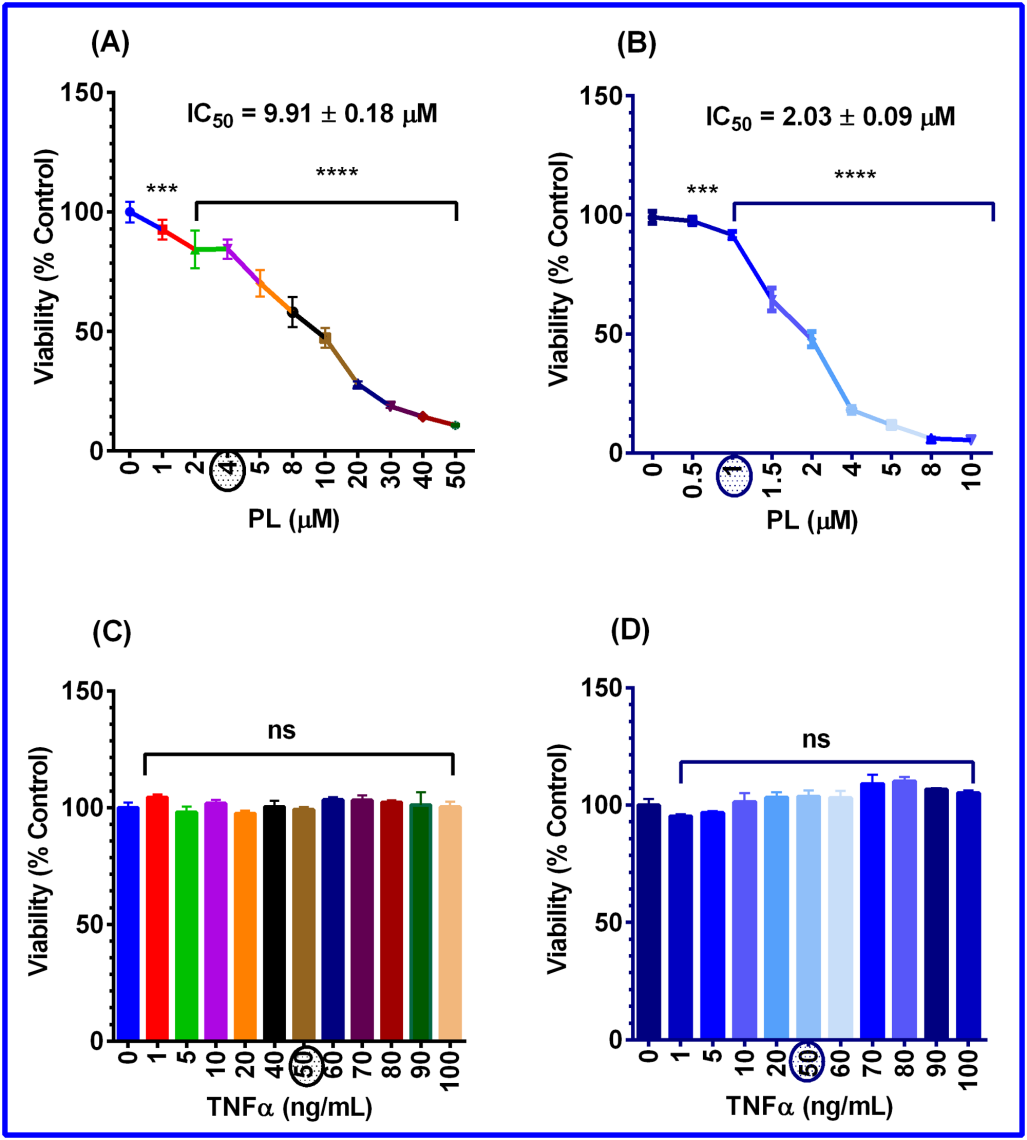
The effect of plumbagin (PL) and TNF-α on the viability of MM-231 and MM-468 cell lines. Cells were treated for 24 h with PL in concentration ranges of 1–50 μM **(A)** and 0.5–10 μM **(B)** in MM-231 and MM-468 cells, respectively. Both cell lines, MM-231 **(C)** and MM-468 **(D)**, were treated with TNF-α in a 0–100 ng/mL concentration range. On the x-axis, the circles represent the working concentrations to be used in the study. The percentages of cell survival compared to the control were calculated. The data points are expressed as the mean ± SEM of three independent studies, n = 4. The significance of the difference between the control and treated groups was determined using the one-way ANOVA followed by the Bonferroni’s multiple comparisons. ***p <0.001, ****p <0.0001, and nonsignificant (ns).

To determine the anticancer effects of PL on TNBC cells, we first examined the cytotoxicity of PL in both MM-231 and MM-468 cell lines. As shown in Fig 1A and 1B, a highly significant effect (p < 0.0001) was found in different PL concentration ranges tested in MM-231 and MM-468 cells. The obtained data indicate that PL was 5-fold more effective in MM-468 cells (IC_50_ = 2.03 ± 0.09 μM) than in the MM-231 cell line (IC_50_ = 9.91 ± 0.18 μM). Additionally, we identified the optimum concentration of the proinflammatory cytokine TNF-α to stimulate inflammatory cytokines in both cell lines. Fig 1C and 1D show that increasing concentrations (1–100 ng/mL) of TNF-α had no significant effect on the cell lines examined compared to the control. From these results, as well as from previous reports [40], we selected 50 ng/mL TNF-α as a working concentration in the study.

**Fig 2:**
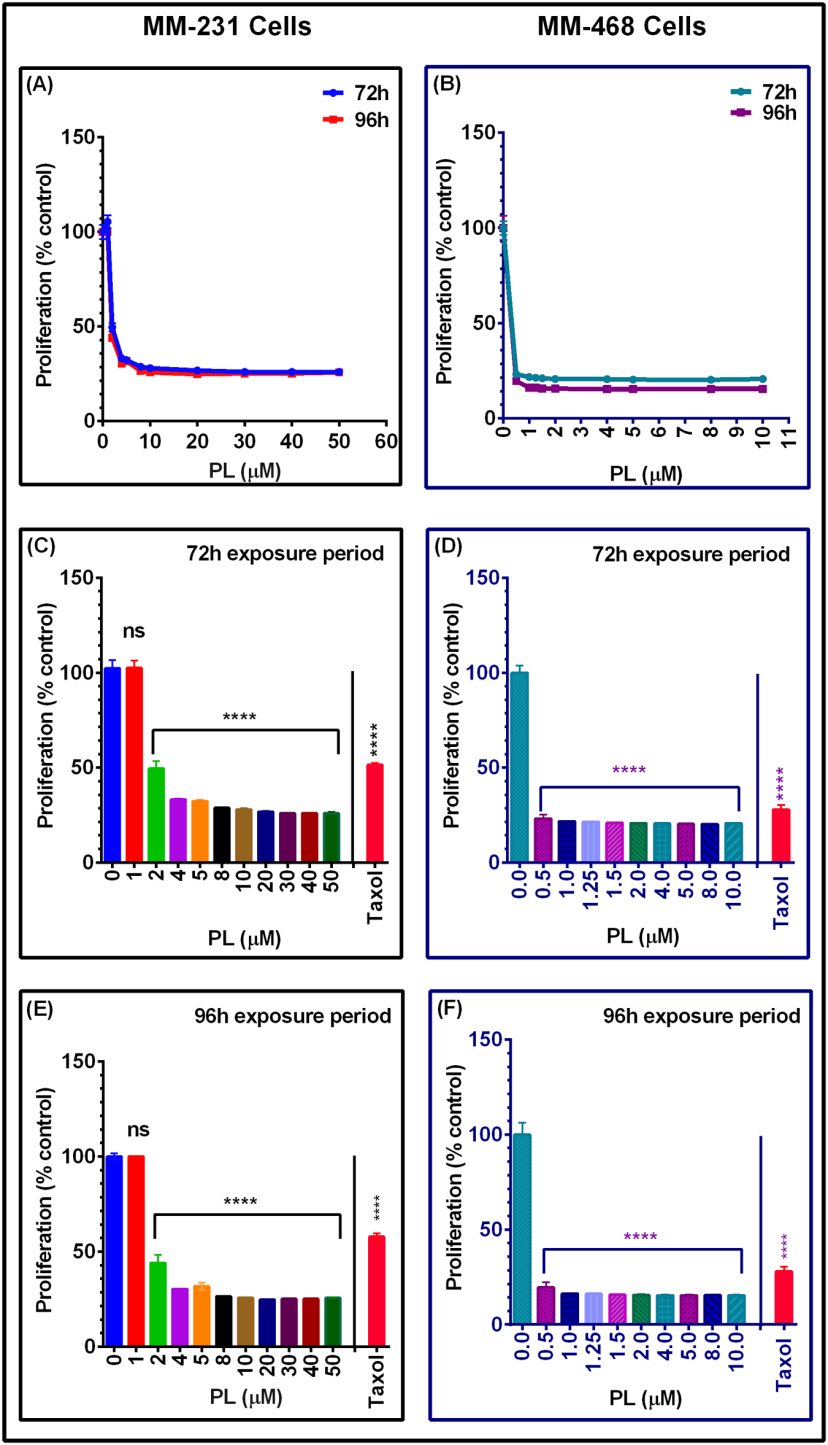
The effect of PL as an antiproliferative agent in MM-231 and MM-468 TNBC cells. Cells were incubated for 72 or 96 h with PL in concentration ranges of 0–50 or 0–10 μM, respectively (A and B). For the 72-h (C and D) or 96-h exposure periods (E and F), Taxol^®^ (1 μM) was applied to both cell lines. Each data point represents the mean ± SEM of three independent experiments, n = 4. The significance of the difference between 72-h vs. 96-h exposure period was calculated using two-way ANOVA, while that between the control vs. treatment was established with one-way ANOVA, and both were followed by Bonferroni’s multiple comparisons. **** p< 0.0001 indicates the statistically significant difference between the control and treated groups after the 72-h or 96-h exposure periods.

The antiproliferative assay was used to evaluate the inhibitory effect of PL on the proliferation of MM-231 and MM-468 cells in comparison with the antiproliferative effect of the standard anticancer drug Taxol^®^. Inhibition of TNBC cell proliferation was verified by measuring the metabolic activity and the ability to reduce resazurin after a 72-h or 96-h exposure period. In both cell lines, measurements of the proliferation rate at 72 or 96 h of PL exposure showed significant inhibition compared to the rate in the control cells, but there was no significant difference in cell proliferation inhibition between the two periods of incubation (Fig 2A and 2B) for MM-231 and MM-468 cells, respectively. The data also show that PL significantly inhibited MM-231 and MM-468 cell proliferation (p< 0.0001) at ≥2 μM concentrations in MM-231 cells (Fig 2C and 2E) and ≥0.5 μM concentrations in MM-468 cells (Fig 2D and 2F). Additionally, the viability study data indicate that PL was highly potent when tested in MM-468 cells. During the 96-h exposure period, 0.5 μM PL decreased the proliferation rate by 80% as compared with a 70% decrease in cells treated with 1 μM of Taxol^®^ (Fig 2F). Considering these data together with the nontoxic effect of 0.5 μM PL on MM-468 cells (Fig 1B), we excluded the probability that the decrease in proliferation was due to cytotoxicity.

**Fig 3:**
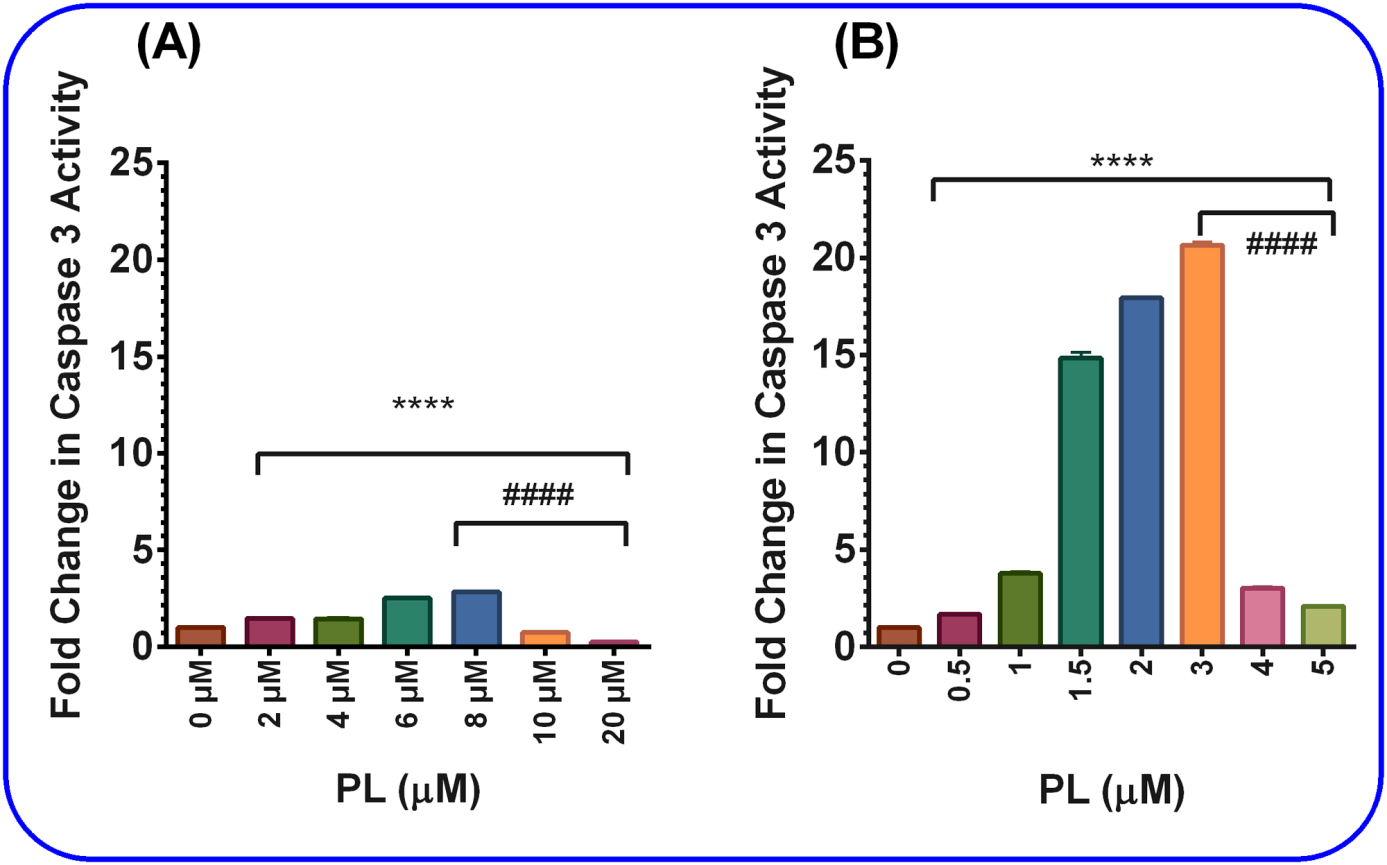
The effect of PL on caspase-3 activation. PL caused caspase-3 activation in both MM-231 and MM-468 cell lysates after cell treatment with PL for 24 h in concentration ranges of 0–20 μM for MM-231 cells (A) or 0–5 μM for MM-468 cells (B). The data represent two independent studies with n = 3 and are expressed as a fold-increase compared to the control. **** p *<* 0.0001 indicate the highly significant difference between the control vs. treated cells. Likewise, ^####^ p *<* 0.0001 between the PL concentration-induced highest caspase level and caspase levels at further increasing PL concentrations in both cell lines.

The PL effect on different TNBC cell lines was tested in both MM-231 and MM-468 cells to determine whether apoptosis mediates the antiproliferative effect of PL. The change in caspase-3 activity, as a marker for apoptosis and subsequent cell death, was assessed in both cell lines relative to that in the control cells. After the 24-h treatment period, a significant gradual increase in caspase-3 level (p< 0.0001) in a dose-dependent manner was detected in both cell lysates, reaching its maxima at 8 μM in MM-231 and 3 μM in MM-468 cells. After that, a dramatically significant decrease in caspase-3 activities (p< 0.0001) with increasing PL concentrations was detected in both cell lines. Noticeably, 8 μM PL caused a three-fold increase in caspase-3 activity in MM-231 cells (Fig 3A), while 3 μM PL caused a 20-fold increase in caspase-3 activity in MM-468 cells (Fig 3B). These results confirmed that apoptosis was taking place in both TNBC cell lines and indicated a higher sensitivity of MM-468 cells to PL.

**Fig 4:**
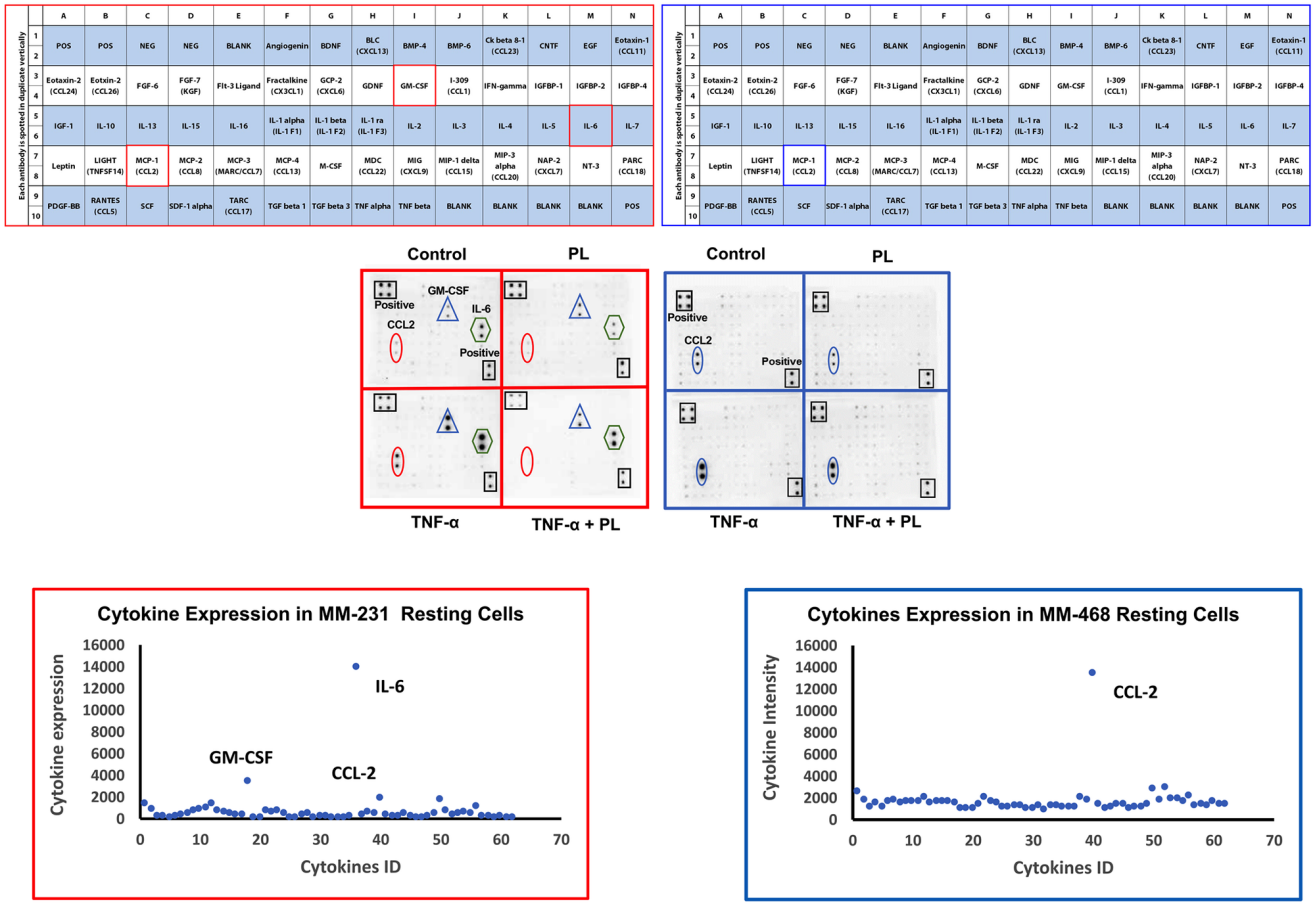
The effect of PL on different cytokine expression in TNF-α-activated MM-231 and MM-468 TNBC models. Microarray map AAH-CYT-6 was used to assess chemokine/cytokine expression in the cell-free supernatants (A1 and B1). For each cell line, four blots represent the supernatants of the following cells: control, 4 μM PL (MM-231 cells) or 1 μM PL (MM-468 cells), TNF-α (50 ng/mL)-treated and, lastly, PL and TNF-α-treated. Microarray chemiluminescence detected the changes in cytokine expression in MM-231 and MM-468 cells (A2 and B2). Panels A3 and B3 represent highly expressed cytokines in resting cells. For MM-231 cells, the most-attenuated cytokines are CCL2, GM-CSF, and IL-6. However, CCL2 was the only impacted cytokine in MM-468 cells.

To further assess the PL effects on TNBC cells, we measured the expression of cytokines secreted into both MM-231 and MM-468 cell-free supernatants in the presence and the absence of PL and TNF-α. Optimum concentrations of PL and TNF-α were established for cytokine expression assays according to the viability study results and previous studies [40, 41]. Both cell lines were stimulated with 50 ng/mL TNF-α, in addition to the low doses of PL that slightly affected cell viability (4 μM in MM-231 cells or 1 μM in MM-468 cells). Two different sets of AAH-CYT blots, AAH-CYT-6 and AAH-CYT-7, were used. However, the data obtained from AAH-CYT-7 blot were not significant (not presented). Three cytokines were highly expressed in the resting MM-231 cells: interleukin 6 (IL-6), granulocyte-colony stimulating factor (GM-CSF), and CCL2 (Fig 4A1–4A3). In comparison, CCL2 was the only dominant and highly expressed cytokine in MM-468 cells (Fig 4B1–4B3). Indeed, the data show that CCL2 was highly expressed in both cell lines, although its expression in MM-468 cells was 7-fold higher than in MM-231 cells, as shown in Fig 4A3 and 4B3. High expression of the cytokines was visually observed in the arrays as higher spot intensities upon treatment of both cell lines with 50 ng/mL TNF-α (Fig 4A2 and 4B2). Moreover, PL (4 and 1 μM in MM-231 and MM-468 cells, respectively) attenuated spot intensities.

**Fig 5:**
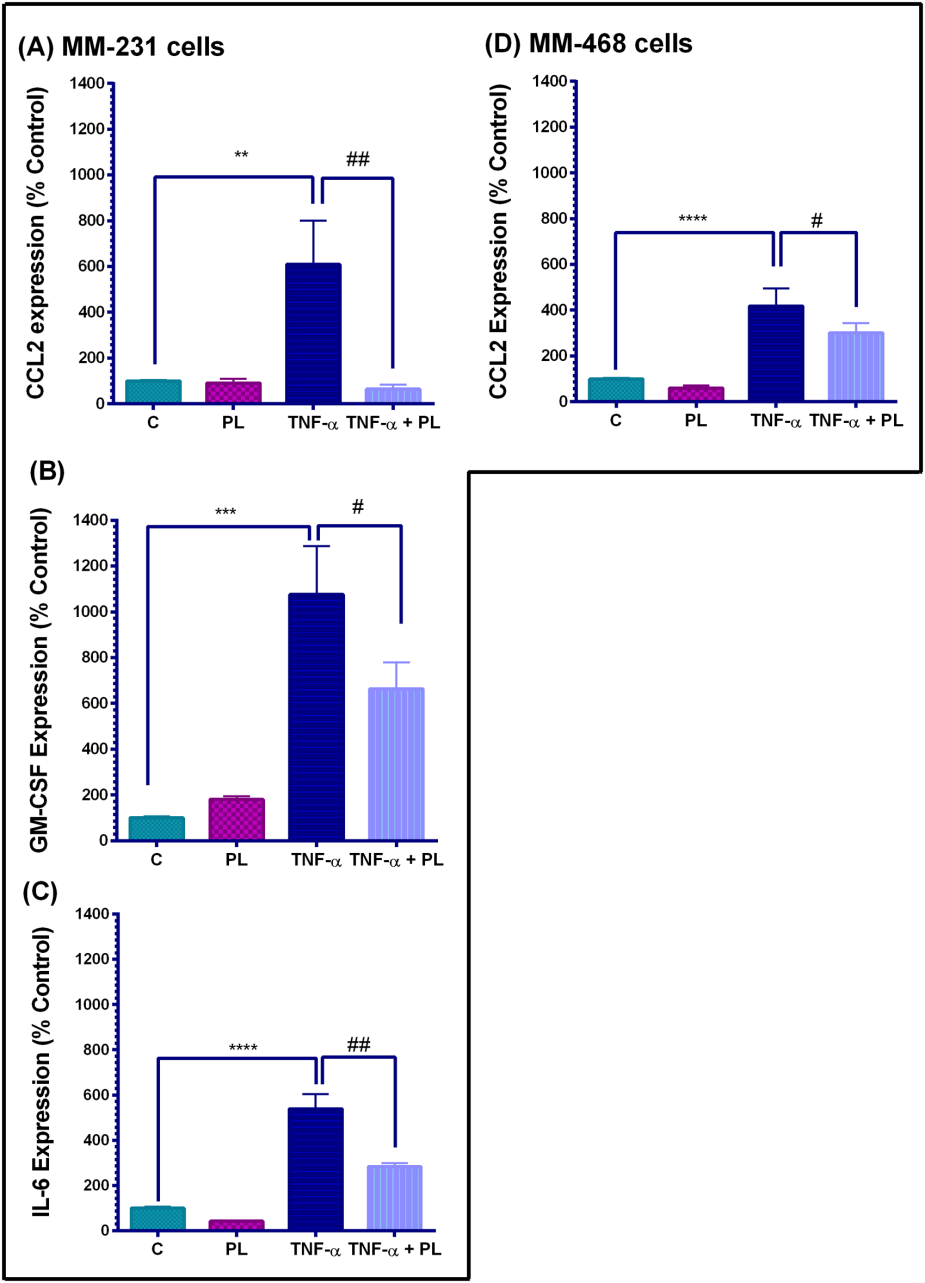
Cytokine array quantification in TNF-α-stimulated TNBC cell lines. The effect of PL on the expression of CCL2, GM-CSF, and IL-6 in MM-231 cells (A-C), and CCL2 expression in MM-468 cells (D). The normalized data for each cell line show cytokine expression in four sets of experimental cell supernatants; control cells, PL-treated cells (4 or 1 μM PL, in MM-231 and MM-468 cells, respectively), TNF-α-stimulated cells (50 ng/mL), and co-treated cells (TNF-α +PL). The data points are expressed as the mean ± SEM of two independent studies, and the intensities are expressed as percent relative to control. The significant difference between the resting and TNF-α-activated cell groups (*), was determined by an unpaired t-test; the same for TNF-α stimulated vs. TNF-α+PL-treated cells (#). Significance is considered at **p < 0.01, ***p < 0.001, ****p < 0.0001, ^#^p < 0.05 and ^##^p < 0.01.

Cytokine array quantification in TNF-α-stimulated TNBC cell lines is presented in Fig 5. Indeed, TNF-α significantly increased the expression of specific cytokines to varying degrees (p<0.01—p<0.0001). In the meantime, no significant difference was found between resting vs. PL-treated cells in both cell lines. In MM-231 cells, the simultaneous presence of PL and TNF-α significantly attenuated three highly TNFα-induced proinflammatory cytokines (Fig 5A–5C), and the CCL2 expression was inhibited by as much as 90% (p < 0.01) (Fig 5A). On the other hand, treatment of TNF-α-stimulated MM-468 cells with PL attenuated the level of highly expressed chemokine CCL2 by 30% (p < 0.05) (Fig 5D). Therefore, these data show different effects of PL on the same CCL2 cytokine in the TNBC cells different by racial origin. Moreover, cytokine GM-CSF exceeded the 10-fold increase compared with the resting MM-231 cells, thus being the most-upregulated cytokine by TNF-α. PL significantly inhibited this cytokine by 40% (p < 0.05) (Fig 5B) and attenuated the highly expressed IL-6 by 50% (p < 0.01) (Fig 5C).

**Fig 6:**
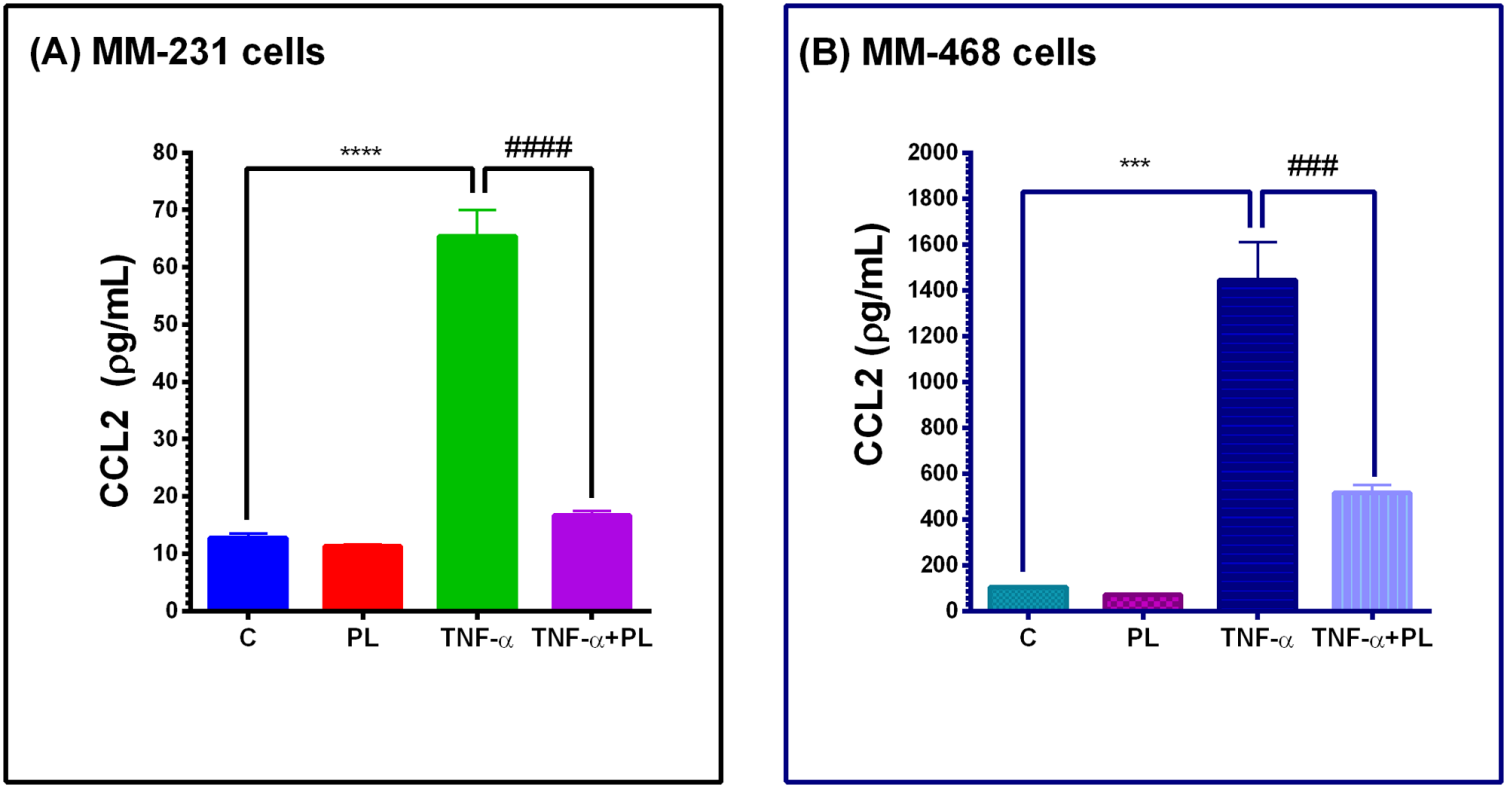
The effect of PL on CCL2 release in TNF-α-treated MM-231 (A) and MM-468 (B) cells. For each cell line, chemokine quantification (pg/mL) was done in four sets of samples. The data points are expressed as the mean ± SEM of two independent studies. The significant difference between the resting vs. TNF-α -activated groups was determined by an unpaired t-test (*), as the same for TNF-α -treated vs. TNF-α + PL-treated cells (#). Significance is considered at ****p < 0.0001, ***p < 0.001, ^####^p < 0.0001 and ^###^p < 0.001.

To further validate the data, two independent ELISA studies were conducted for MM-231 and MM-468 cells to quantify CCL2 attenuation by PL (Fig 6A and 6B). In both cell lines, a highly significant increase was found in resting vs. TNF-α-treated cells (p < 0.0001 and p < 0.001 for MM-231 and MM-468, respectively) as well as in TNF-α vs. TNF-α + PL-treated cells (p < 0.0001 and p < 0.001 for MM-231 and MM-468, respectively). Overall, the data obtained were consistent with the array findings, and CCL2 was attenuated by 75% and 35% in MM-231 and MM-468 cells, respectively.

**Fig 7:**
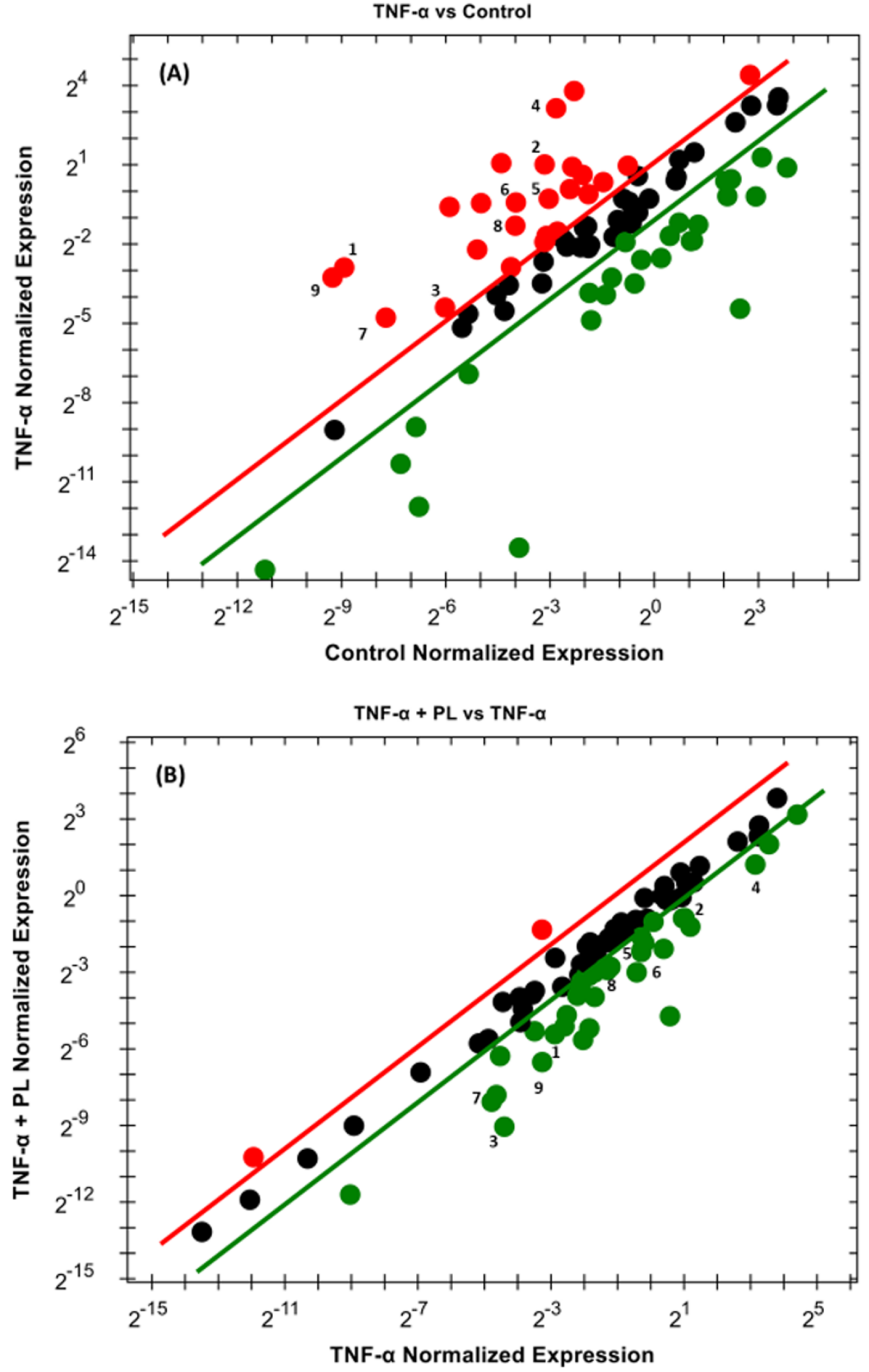
Scatter plot for MM-231 cells. Normalized expression of mRNA target genes for the control vs. TNF-α-stimulated cells (A) and TNF-α-stimulated vs. cotreated cells (B). The plot image shows the following changes in target gene expression based on the threshold set: upregulation, red dots; downregulation, green dots; and no change, black dots. The most downregulated genes are enumerated from 1–9 as follows: *BCL2A1*, *BIRC3*, *CCL2*, *ICAM1*, *IKBKE*, *IL1β*, *LTA*, *TLR2*, and *TNF*.

**Fig 8:**
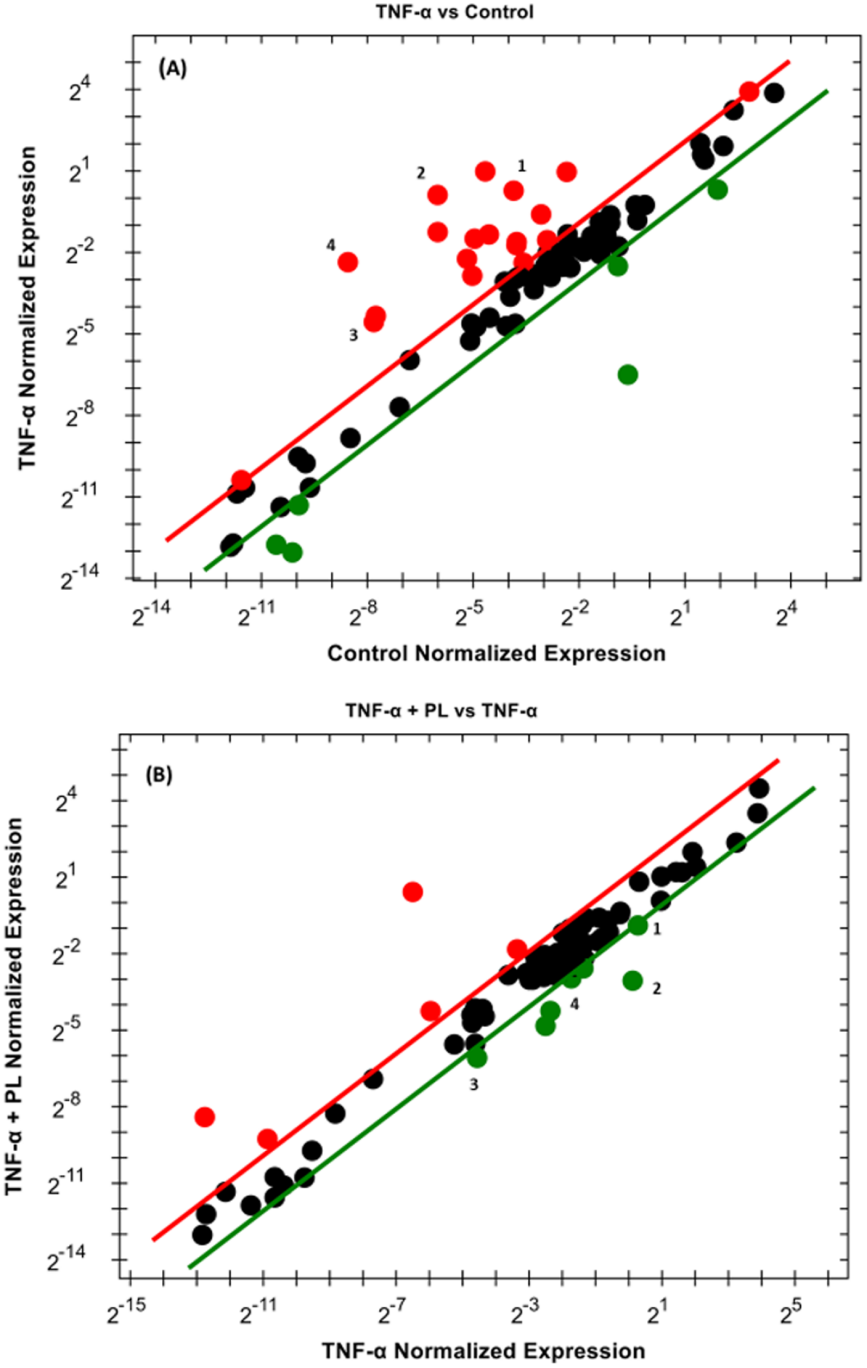
Scatter plot for MM-468 cells. Normalized mRNA expression of target genes for the control vs. TNF-α stimulated cells (A) and TNF-α vs. TNF-α + PL (B). The plot image shows the following changes in target gene expression based on the threshold set: upregulation, red dots, downregulation, green dots, and no change, black dots. The most-downregulated genes are enumerated from 1–4 as follows: *BIRC3*, *CCL2*, *TLR2*, and *TNF*.

To elucidate the mechanism by which PL attenuates CCL2 release in TNF-α-treated TNBC cell models, RT-PCR was performed to investigate the NF-қB signaling pathway. In both cell lines, the normalized mRNA expression in the NF-қB signaling pathway indicates the cells’ response to either TNF-α or TNF-α + PL. Here, we present only the genes that are significantly repressed by PL. The data show that PL significantly downregulated four genes in both cell lines (*BIRC3*, *CCL2*, *TLR2*, and *TNF*). However, in MM-231 cells five more genes were downregulated (*BCL2A1*, *ICAM1*, *IKBKE*, *IL1β*, and *LTA*) (Table 1). The elevated expression of these genes by TNF-α is visually recognized as red dots in MM-231 and MM-468 cells (Figs 7A and 8A, respectively). In the presence of TNF-α + PL, the same genes appear in green as an indication of PL potency to downregulate their expression (Figs 7B and 8B) in MM-231 and MM-468 cells, respectively.

**Table 1:** mRNA gene expression changes in MM-231 TNBC. The left side of the table presents the NF-қB-controlled genes that are upregulated (+ fold-changes) by 50 ng/mL TNF-α. In contrast, the right side presents expression of genes that are downregulated (- fold-changes) by 4 μM PL. p < 0.05 is considered statistically significant.

Control vs. TNF-α			TNF-α vs. TNF-α + PL		
Target gene	Fold changes	p-value	Target gene	Fold changes	p-value
***BCL2A1***	60.29	< 0.0001	***BCL2A1***	-5.36	0.001
***BIRC3***	3.05	0.011	***BIRC3***	-1.80	0.002
***CCL2***	2.90	0.039	***CCL2***	-25.58	0.007
***ICAM1***	6.21	0.006	***ICAM1***	-5.47	0.007
***IKBKE***	1.57	0.378	***IKBKE***	-6.05	0.010
***IL1β***	11.79	0.003	***IL1B***	-9.50	0.004
***LTA***	5.67	0.005	***LTA***	-6.98	0.005
***TLR2***	2.43	0.116	***TLR2***	-2.76	0.040
***TNF***	56.13	< 0.0001	***TNF***	-9.25	0.0002

**Fig 9:**
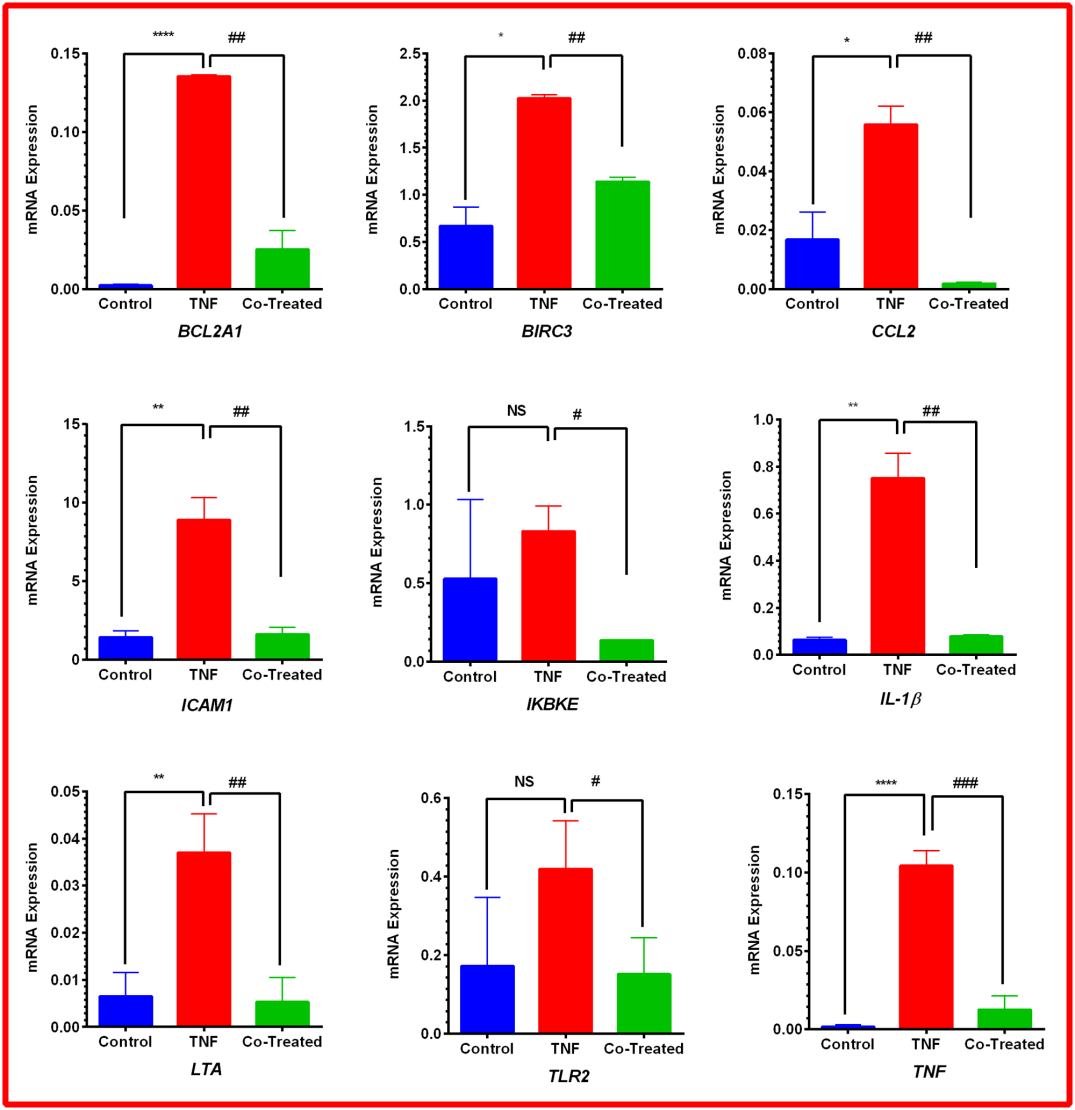
Gene expression quantification in MM-231 cells. Normalized data show a significant increase in seven genes in the TNF-α-stimulated MM-231 cells vs. resting cells. On the other hand, nine genes were significantly repressed in cotreated cells. The data points are expressed as the mean ± SEM of three independent studies. The significance of the difference was determined by an unpaired t-test between the resting vs. TNF-α-activated cells (*), as well as TNF-α-treated vs. TNF-α + PL-treated cells (#). Significance is considered at *p < 0.05, **p < 0.01, ****p < 0.0001, #p < 0.05, ##p < 0.01, and ###p < 0.001 or the difference was nonsignificant (ns).

**Fig 10:**
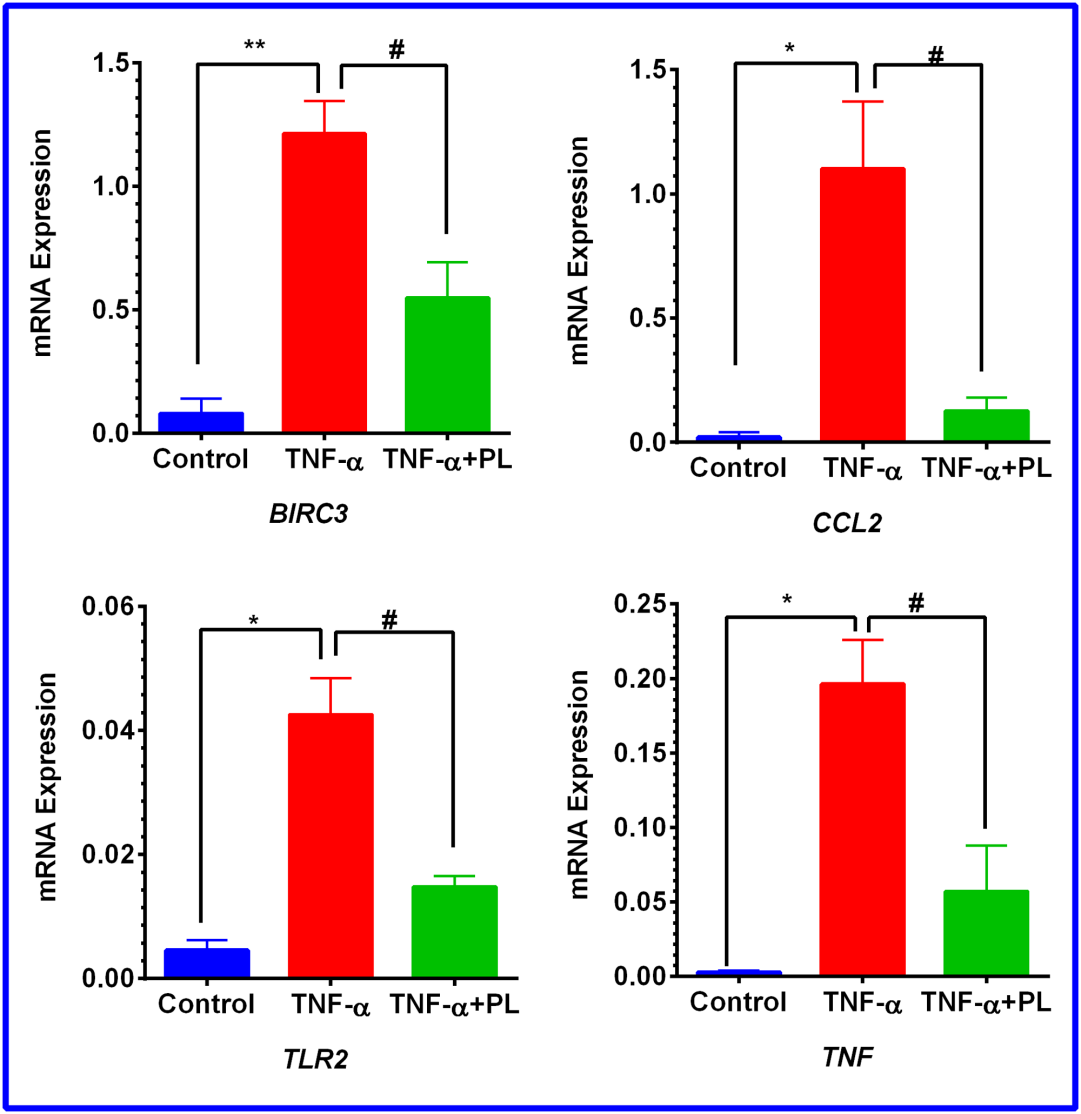
Gene expression quantification in MM-468 cells. Normalized data show a significant increase in four genes in TNF-α-stimulated cells. On the other side, significant gene repression was observed in cotreated cells. The data points are expressed as the mean ± SEM of two independent experiments. The significant difference between resting and TNF-α-activated groups was determined by an unpaired t-test (*), as the same for TNF-α-treated vs. TNF-α + PL-treated cells (^#^). Significance is considered at *p < 0.05, **p < 0.01, and #p < 0.05.

Notably, among these nine upregulated genes in MM-231 cells, seven genes were significantly upregulated by TNF-α, giving the highest significant (p < 0.0001) fold-increases in *BCL2A1* (60.29) and *TNF* (56.13) expression (Table 1 and Fig 9). On the other hand, in MM-468 cells, *TNF* and *CCL2* genes were significantly upregulated (p < 0.05) with 70.09 and 53.05-fold increases, respectively (Table 2 and Fig 10). Interestingly, in the TNF-α-treated cells, the expression of *TNF* and *CCL2* genes was higher in MM-468 cells than in MM-231 cells; however, the fold-decrease was always higher in MM-231 cells. Noticeably, the fold-increase in *TNF* gene in MM-468 cells was 70.09 vs. 56.13 in MM-231 cells. However, MM-231 cells were more affected by PL, with the expression of *TNF* decreasing 9.25-fold compared with 3.44-fold in MM-468 cells. Similarly, the fold-increase in *CCL2* was 2.90 vs. 53.05 in MM-231 and MM-468 cells, respectively. Nevertheless, a dramatic decrease in expression of this gene was found in MM-231 cells compared to MM-468 cells (25.58 vs. 8.75, respectively). Nevertheless, whereas a nonsignificant TNF-α-induced upregulation was observed for two genes, *IKBKE* and *TLR2* (Table 1 and Fig 9), PL significantly inhibited their mRNA expression (p < 0.05).

**Table 2:** mRNA gene expression changes in MM-468 TNBC. The left side of the table presents the NF-қB-controlled genes that are upregulated (+ fold-changes) by 50 ng/mL TNF-α. In contrast, the right side presents expression of genes that are downregulated (- fold-changes) by 1 μM PL in MM-468 cells. p < 0.05 is considered statistically significant.

Control vs TNF-α			TNF-α vs TNF-α + PL		
Target gene	Fold changes	p-value	Target gene	Fold changes	p-value
***BIRC3***	14.92	0.008	***BIRC3***	-2.211	0.040
***CCL2***	53.05	0.030	***CCL2***	-8.750	0.038
***TLR2***	9.28	0.013	***TLR2***	-2.870	0.024
***TNF***	70.09	0.012	***TNF***	-3.441	0.044

## Discussion

In the cancer cells, targeting inter- and intra-cellular signaling pathways involved in proliferation, inflammation, and apoptosis is a well-suited approach to the development of TNBC anticancer agents. NF-қB is a critical component of cancer proliferation and metastasis [14]. It regulates the genes linked to tumor proliferation [42], plays a proapoptotic role [14] and mediates the expression of antiapoptotic genes [43].

The present study elucidated the anticancer mechanism of the natural quinone PL. This compound attenuated the TNF-α-mediated increase in expression of CCL2 and other cytokines through the downregulation of the mRNA expression of specific genes involved in the NF-қB pathway (Figs 4–10). The data obtained show the differences in the antiproliferative, apoptotic, and cytotoxic effects of PL with a higher response in MM-468 cells than in MM-231 TNBC cells (Figs 1–3).

Under normal conditions, the secretion and release of signals for maintaining homeostasis are precisely controlled. However, deregulation of these signals by triggering distinctive gene expression is the major key to uncontrolled cell proliferation of cancer tissue [44]. Furthermore, the products of these genes, including caspases, are critical in the regulation of apoptosis. Caspase-3, a downstream caspase has been shown to play a critical role in the terminal execution segment of apoptosis induced by diverse stimuli [45, 46]. Previous studies have reported that PL can affect cellular proliferation, carcinogenesis, and radio-resistance, and all are regulated by activation of the transcription factor NF-қB. Consistent with the current data, PL exhibited significant antitumor effects by inducing apoptosis and decreasing proliferation in both MM-231 and MM-468 TNBC cells, yet MM-468 was more sensitive to PL in the proliferation and apoptosis experiments (Figs 2 and 3, respectively).

It is known that chemokine CCL2 is released into the tumor microenvironment by endothelial cells and fibroblasts [47] to activate lymphocytes and macrophages [48]. Additionally, CCL2 is overexpressed in several malignant tumors, including MM-231-tumor xenograft for TNBC models [49, 50]. Indeed, the reported overexpression of CCL2 was linked to the decreased survival of TNBC patients [51]. Additionally, the reduction of CCL2 expression after antibody treatment and genetic modification [52] led to reduced metastasis and improved survival [53]. Moreover, targeting CCL2 decreased the recruitment of both tumor-associated macrophages (TAMs) and myeloid-derived suppressor cells (MDSCs) to the tumor site and reduced primary tumor growth [54]. Downregulation of CCL2 was also reported to enhance tumor cell apoptosis [55] and block the recruitment of inflammatory monocytes, inhibit lung metastasis of BC cells and prolong the survival of tumor-bearing mice [52] and TNBC patients [56]. Thus, the reported wide distribution and increase in the expression of this cytokine in different cancers, particularly TNBC, indicate its significant role in cancer aggressiveness.

The mechanism by which CCL2 impacts on cancer is reported to be widely diverse. The interaction between CCL2 and its primary receptor CCR2 is crucial for recruiting large numbers of monocytes to the tumor tissue [51]. This interaction can lead to the enhancement of tumor growth and survival [57], initiation of tumor angiogenesis [56], inhibition of antitumor immunity [58], and finally, promotion of tumor invasion and metastasis [59]. Moreover, CCL2 regulates cancer stem cells through an abnormal Notch activation in various tissues [60], which is also fundamental to BC [61, 62].

Upon exposure to TNF-α, overexpressed extracellular CCL2 was dramatically and significantly amplified in both cell lines (Figs 4–6), an observation consistent with previous reports [63, 64]. It has been reported that TNF-α is highly expressed in BC patients [18]. However, its function as tumor necrosis or tumor promoting factor is related to whether it is endogenous or is administered in a high dose, respectively [65]. When adding the quinone PL to our TNF-α-activated TNBC cell models, CCL2 expression was significantly and differentially reduced. At nontoxic concentrations of PL, despite a highly aggressive nature of TNBC in African-Americans, the MM-468 model showed lower CCL2 inhibition compared to MM-231 (Figs 5 and 6). The presence of over-expressed and conceivably stimulated CCL2 in both TNBC racially disparate models emphasizes its crucial role in TNBC aggressiveness and, hence, highlights involvement of the PL suppression mechanism in TNBC treatment, particularly, of CCL2 in the MM-231 model that was dramatically suppressed (Figs 5A and 6A).

In BC, NF-қB activation increases the expression of CCL2 [66] and other inflammatory cytokines/chemokines/interleukins, including IL-1, IL-6, IL-8, TNF-α, and CXCL8, as well as cyclooxygenase 2 (COX2) and nitric oxide synthase (NOS) [67, 68]. Moreover, NF-қB influences cancer radio- and chemo-resistance [69]. Our results show that upon exposure to TNF-α, the number of genes significantly upregulated by NF-қB was higher in MM-231 than MM-468 cells, even with the previously reported poor sensitivity of MM-231 cells at the same TNF-α concentration as in our study [40].

The profile of NF-қB-regulated genes in our data indicates the racial differences in BC (Table 1 and Figs 7–10). Consistently, in TNF-α + PL-treated MM-468 cells, the reduction in the TNFα-induced increase in gene expression was always lower than in their counterpart MM-231 cells (Table 1). In the presence of TNF-α, PL was able to significantly downregulate nine genes in MM-231 cells (*BCL2A1*, *BIRC3*, *CCL2*, *ICAM1*, *IKBKE*, *IL1β*, *LTA*, *TLR2*, and *TNF*) (Table 1). These genes are involved in other inter/intracellular mechanisms controlling apoptosis, proliferation, invasion, and metastasis. The MM-468 cell line had only four genes downregulated (*BIRC3*, *CCL2*, *TLR2*, and *TNF*) (Table 2). These data indicate that PL is more potent in affecting the CA MM-231 cells through the NF-қB-downregulated genes than the AA MM-468 cell model of TNBC.

PL dramatically decreased the TNF-α-stimulated *TNF* and *LTA* gene expression only in MM-231 cells (Table 1). Together, the TAM-derived *TNF* and *LTA* are involved in breast carcinogenesis [70, 71]. The *LTA* gene is a member of the TNF family that activates NF-қB [72] and facilitates inflammation [73]. Mainly, *LTA* was considered as an important biomarker of BC predisposition, and its gene polymorphism was linked to an elevated risk of BC in AA [74], and CA [75].

In both cell lines, TNF-α upregulated *BIRC3* expression, which was significantly suppressed by PL. *BIRC3* is a member of the family of inhibitors of apoptosis (*IAP*), and NF-қB is involved in its gene transcription [76]. The *IAP* genes can, in turn, drive and potentiate NF-қB activity [77] and consequently increase CCL2 expression. Particularly, the *BIRC3* gene was reported to regulate the caspase activity [78], apoptosis, cellular differentiation, and proliferation [79]. Rationally, its repressor PL showed an antiproliferative mechanism by inducing G2-M arrest and autophagy in BC cells MCF-7 and MM-231 [80]. Thus, our data highlight the importance of PL in targeting *BIRC3* to indirectly attenuate CCL2 expression and inhibit proliferation in both TNBC cell lines.

Additionally, *TLR2* was equally downregulated by PL in both cell lines (~3-fold decrease). Our results are consistent with a previous study that linked *TLR2* inhibition to the reduction of constitutively active NF-қB, inhibition of the cytokine IL-6 production and decrease in cell proliferation [14]. *TLR2/4* and their coactivator CD14 are highly expressed in ER^-^ tumors [81] and are involved in NF-қB activation and an increase in metastasis [82, 83]. Thus, our data support recently proposed *TLR2* inhibition in fighting BC [84].

Notably, in MM-231 cells, more genes were significantly downregulated by PL: *BCL2A1*, *ICAM1*, *IKBKE*, *and IL-1β*. These genes are overexpressed in TNBC cells and involved in the NF-қB pathway activation and CCL2 release. The *BCL2A1* gene is critical for drug resistance [85, 86]. Therefore, targeting *BCL2A1* has shown preclinical promise, either by itself or in combination with other anticancer agents [87], similarly to the *ICAM1* gene [88]. Downregulation of the *IKBKE* gene was previously reported to attenuate CCL2 release [89] that led to cancer cell death [90]. Additionally, the *IL-1β* gene is linked to the invasiveness of TNBC cells [91], and certainly, downregulation of this gene will reduce cancer cell proliferation, invasion, and metastasis [21].

In summary, the current study sheds light on a novel compound targeting TNBC, among African Americans in particular. This compound impacts MM-231 and MM-468 cells differently. PL attenuated the expression of three cytokines (CCL2, IL-6, and GM-CSF) in MM-231 cells and cytokine CCL2 in MM-468 cells. The data obtained showed the potency of PL to attenuate many regulatory genes involved in the NF-қB pathway, the major factor in releasing CCL2. PL downregulated the expression of four genes in MM-468 cell model shared with MM-231 cells (*BIRC3*, *CCL2*, *TLR2*, and *TNF*), and five more distinct genes were downregulated in the MM-231 cells (*BCL2A1*, *ICAM1*, *IKBKE*, *IL1β*, and *LTA*). This finding explains the dramatic downregulation of CCL2 in MM-231 cells compared to their counterpart in MM-468 cells. However, PL was more potent in the MM-468 than in MM-231 cells in decreasing cell viability and proliferation and inducing apoptosis by increasing caspase-3. These findings might indicate the existence of two different anticancer mechanisms of PL in TNBC cells, a mechanism that tends to have a more effective apoptotic action in MM-468 cells, and another that has a more NF-қB-induced CCL2 inhibitory action in MM-231 cells. In conclusion, the data obtained in this study indicate that the quinone compound PL could be an attractive targeting agent for cancer therapy for African American women with TNBC.

## Experimental section

### Materials and reagents

PL (purity 99%), dimethyl sulfoxide (DMSO), 0.25% Trypsin-EDTA solution, Alamar Blue^®^ (solution of resazurin fluorescence dye), Taxol^®^ (paclitaxel), and lipopolysaccharides from Escherichia coli O111: B4 (LPS) were purchased from Sigma-Aldrich (St. Louis, MO, USA). Cell culture flasks and plates, Dulbecco’s Modified Eagle Medium (DMEM) and fetal bovine serum (FBS) were purchased from VWR International (Radnor, PA, USA). Penicillin/streptomycin and DPBS were obtained from Atlanta Biologicals (Atlanta, GA, USA). Human Cytokine Antibody Array Kit (Cat # AAH-CYT-1000), Human MCP-1 ELISA kit (Cat # ELH-MCP1) and TNF-α were purchased from RayBiotech (Norcross, GA, USA). SsoAdvancedTM Universal SYBR^®^ Green Supermix and NF-қB Signaling Pathway (SAB Target List) H96 were purchased from Bio-Rad (Bio-Rad, Hercules, CA). DNA-free^™^ Kit (Cat # AM1907) and EnzChek^®^ Caspase-3 Assay were purchased from Life Technologies Inc. (Grand Island, NY, USA).

### Cell culture

TNBC cell models, MM-231 and MM-468, were purchased from American Type Culture Collection (ATCC). Both cell lines were grown in 75-cm TC flasks at 37°C in humidified 5% CO_2_ incubator and were subcultured, as needed, with trypsin/EDTA. The DMEM growth medium contained 4 mM L-glutamine and was supplemented with 10% heat-inactivated FBS (v/v), and 1% penicillin/streptomycin salt solution (100 U/mL and 0.1 mg/mL, respectively). The DMEM experimental medium was phenol-free and supplemented with 2.5% heat-inactivated FBS.

### Cell viability assay

In this experiment, cells were incubated overnight in the experimental media at a density of 5×10^4^ cell/well in 96-well plates. Both types of cells were treated for 24 h with TNF-α (0–100 ng/mL) or PL (concentration ranges of 0–50 μM in MM-231 or 0–10 μM in MM-468 cells). PL was solubilized in DMSO, while TNF-α was dissolved in cell culture water. Control wells were treated with DMSO at the highest used concentration (< 0.1%). Equivalently treated wells without cells were used as blanks. In this assay, Alamar Blue^®^ was used to determine cell viability at a concentration level of 10% v/v and an incubation time up to 6 h. The fluorescent fuchsia—reduced resazurin dye was measured at an excitation/emission of 530/590 nm using a Synergy HTX Multi-Reader (BioTek, USA).

### Cell proliferation study

The effects of PL on cell proliferation were determined for MM-231 and MM-468 TNBC cells based on the dose-response viability study concentrations [92] using Alamar Blue^®^. Briefly, cells were plated at an initial density of 5×10^3^ cell/well in 96-well plates and incubated overnight. The cells were treated for 72 or 96 h with PL at concentrations ranging between 0–50 μM or 0–10 μM in MM-231 or MM-468 cells, respectively, in a final volume of 200 μL/well. Control cells were exposed to DMSO at a concentration < 0.1%. Taxol^®^ was used as a positive control only at 1 μM concentration level [93], and equivalent wells without cells were used as a blank. Proliferation was measured after the predesigned experimental period by adding Alamar Blue^®^ to each well at 10% v/v and incubating for an additional 4 h. The plates were read at an excitation/emission of 530/590 nm using a Synergy HTX Multi-Reader (BioTek, USA).

### Caspase-3 apoptosis study

The apoptotic effect of PL was determined in MM-231 and MM-468 cells using the EnzChek^®^ Caspase-3 Assay kit. The assay detects apoptosis by assessing the increase in caspase-3 activity. Briefly, each cell line was seeded at an initial concentration of 1×10^6^ cell/well in 6-well plates and incubated overnight. The tested concentrations were determined based on the IC_50_ values of the dose-response viability curve for each cell line. After 24 h, cells were treated with PL at concentrations ranging between 0–20 or 0–5 μM in MM-231 or MM-468 cells, respectively, in a final volume of 3 mL/well experimental media. Control cells were exposed to DMSO at a concentration < 0.1%. After the 24 h incubation period, treated cells from each well were harvested, pelleted, and washed in PBS. Cell pellets were resuspended in 50 μL lysis buffer (200 mM Tris, pH 7.5, 2 M NaCl, 20 mM EDTA, 0.2% Triton^™^ X-100) for 30 min on ice followed by centrifugation for 5 min at 5,000 × g to pellet the debris. Lastly, 50 μL of each of the sample supernatant and the reaction buffer-substrate working solution (50 mM PIPES, pH 7.4, 10 mM EDTA, 0.5% CHAPS, 1 M DTT/5 mM Z-DEVD-R110), were combined in another microplate well for 30 min at RT, and the fluorescence background was determined by using 50 μL of the cell lysis buffer. Fluorescence intensity for each sample was measured (excitation/emission ~342/441 nm) using a Synergy HTX Multi-Reader (BioTek, USA).

### Human cytokine/chemokine protein microarray

For cytokine microarray analysis, four flasks for each cell line were seeded at a density of 10×10^6^ cells/75-cm^2^ TC flask using the same cell culture media. On the day of the experimentation, the media were discarded, and the cells were immediately washed with phenol- free experimental media. Based on our cytotoxicity assay, the MM-231 cells were treated as follows: TNF-α (50 ng/mL), PL (4 μM), or TNF-α + PL (50 ng/mL + 4 μM, respectively). Similarly, MM-468 cells were treated with TNF-α (50 ng/mL), PL (1 μM), and TNF-α + PL (50 ng/mL + 1 μM, respectively). In both experimental sets, control samples were exposed to only DMSO at a concentration < 0.1%. After a 24-h exposure period, the cell-free supernatant of each sample was collected, aliquoted, and stored at -80°C for later use. At the same time, the cells from each sample were pelleted and similarly stored at -80°C for RT-PCR study. For each cell line, a semiquantitative method was established to evaluate chemokine/cytokine expression in the cell culture supernatants using antibody-coated array membranes. The assay was conducted following the protocols supplied with the kits from RayBiotech. Briefly, membranes were carefully placed in the incubation tray and blocked with the provided buffer on a shaker for 30 min at RT. Thereafter, the blocking buffer was decanted, and replaced with 1 mL cell-free supernatant from resting, PL-treated, TNF-α-stimulated or cotreated cells, then placed overnight on a low-speed shaker at 4°C. After 24 h, the media were decanted from each chamber, and the membranes were washed with the kit buffers. Next, 1 mL of freshly constituted biotinylated antibody cocktail was pipetted to each membrane and incubated at RT for 2 h followed by washing with the same wash buffer. The membranes were incubated again for another 2 h with 2 mL of diluted horseradish peroxidase-conjugated streptavidin (HRP-Streptavidin) followed by the final washes. Spot intensities on the blots were evaluated using chemiluminescence detection. The blot images were captured using a Flour-S Max Multiimager (Bio-Rad Laboratories, Hercules, CA, USA), and the spot intensities were obtained with the Quantity-One Software (Bio-Rad Laboratories, Hercules, CA). The Excel-based data analysis was established, using the Human Cytokine Array software C1000 (CODE: S02-AAH-CYT-1000) from RayBiotech.

### Human MCP-1 cytokine ELISA study

Enzyme-Linked Immunosorbent Assay (ELISA) kits were used for both cell lines to confirm the effect of PL on CCL2 chemokine expression detected by the microarray. Briefly, the standard curves, samples, and reagents were prepared at RT. Standards and samples of 100 μL were incubated with the antibody precoated 96-well ELISA plates for 2.5 h. The supernatant was replaced by 100 μL of freshly constituted biotinylated antibody for another hour, then decanted. Streptavidin solution (100 μL) was added for 45 min followed by an addition of 100 μL of the substrate reagent for 30-min incubation. Washes were always performed after each step according to the manufacturer’s protocol. The reaction was terminated by the addition of 50 μL of a stop-solution, and the intensity was measured at 450 nm using a Synergy HTX Multi-Reader (BioTek, USA).

### Reverse transcription-polymerase chain reaction (RT- RCR) array

The current study was performed using the previously -80°C-stored cells as mentioned previously. The total RNA was isolated from each sample using 1 mL Trizol reagent. Thereafter, samples were treated with chloroform (0.2 mL), vortexed, incubated at RT for 2–3 min and centrifuged at 10,000 x g for 15 min at 2–8°C. The aqueous phase was collected, and the RNAs were precipitated by mixing with 0.5 mL of isopropyl alcohol. RNA pellets were then dissolved in nuclease-free water to measure the RNA quantity and quality using a NanoDrop spectrophotometer, and cDNA was synthesized using the iScript^™^ cDNA Synthesis Kit. Following the manufacturer’s protocol, the obtained cDNA was reconstituted in nuclease-free water, and the NF-қB Signaling Pathway 96-well plate was loaded with 10 μL each of the reconstituted cDNA (2.3 ng) and SsoAdvanced^™^ Universal SYBR^®^ Green Supermix, then placed for 5 min in a shaker and centrifuged at 1000 x g for 1 min. The PCR run was established with 39 cycles of denaturation as follows: 30-sec activation at 95°C, 10-sec denaturation at 95°C; 20-sec annealing at 60°C; and 31-sec extension at 65°C using a Bio-Rad CFX96 Real-Time System (Hercules, Ca, USA). All real-time PCR reactions were performed in triplicate for each cell line.

### Statistical analysis

The data were analyzed using the Graph Pad Prism 6.2 Software (San Diego, CA, USA). All data points were obtained from the average of at least two independent studies and expressed as the mean ± SEM. For viability studies, IC_50_ values were determined by nonlinear regression with the R^2^ best fit and lowest 95% confidence interval. The significance of the difference between each control and its related treated groups was determined using the one-way ANOVA followed by Bonferroni’s multiple comparison tests. For the proliferation assay, the statistical analysis was performed with a two-way ANOVA between the 72-h and 96-h exposure periods and followed with Bonferroni’s multiple comparison tests. The significance of the difference was considered at *P < 0.05, **P < 0.01, ***P < 0.001, and ****P < 0.0001. For blots and ELISA studies, Student’s t-test was used to verify the significance of the difference between the control and TNF-α groups, or between the TNF-α and TNF-α +PL groups. Gene expression was analyzed using the CFX 3.1 Manager software (Bio-Rad, Hercules, CA) and verified similarly with Student’s t-test.
